# Associations of polysocial risk score with incident rosacea: a prospective cohort study of government employees in China

**DOI:** 10.3389/fpubh.2023.1096687

**Published:** 2023-05-03

**Authors:** Peng Chen, Ziye Yang, Zhihua Fan, Ben Wang, Yan Tang, Yi Xiao, Xiang Chen, Dan Luo, Shuiyuan Xiao, Ji Li, Minxue Shen

**Affiliations:** ^1^Department of Dermatology, Xiangya Hospital, Central South University, Changsha, China; ^2^Hunan Engineering Research Center of Skin Health and Disease, Xiangya Hospital, Central South University, Changsha, China; ^3^Department of Social Medicine and Health Management, Xiangya School of Public Health, Central South University, Changsha, China; ^4^Hunan Key Laboratory of Aging Biology, Xiangya Hospital, Central South University, Changsha, China; ^5^National Clinical Research Center for Geriatric Disorders, Xiangya Hospital, Central South University, Changsha, China

**Keywords:** rosacea, polysocial risk score, social determinants of health, prospective cohort studies, risk factors

## Abstract

**Background:**

The associations between single risk factors and incident rosacea have been reported, but the effects of social risk factors from multiple domains coupled remain less studied.

**Objectives:**

To quantify the influence of social determinants on rosacea comprehensively and investigate associations between the polysocial risk score (PsRS) with the risks of incident rosacea.

**Methods:**

This was a prospective cohort study of government employees undertaken from January 2018 to December 2021 among participants aged >20 from five cities in Hunan province of China. At baseline, information was collected by a questionnaire and participants were involved in an examination of the skin. Dermatologists with certification confirmed the diagnosis of rosacea. The skin health status of participants was reassessed every year since the enrolment of study during the follow-up period. The PsRS was determined using the nine social determinants of health from three social risk domains (namely socioeconomic status, psychosocial factors, and living environment). Incident rosacea was estimated using binary logistic regression models adjusted for possible confounding variables.

**Results:**

Among the 3,773 participants who completed at least two consecutive skin examinations, there were 2,993 participants included in the primary analyses. With 7,457 person-years of total follow-up, we detected 69 incident rosacea cases. After adjustment for major confounders, participants in the group with high social risk had significantly raised risks of incident rosacea with the adjusted odds ratio (aOR) being 2.42 (95% CI 1.06, 5.55), compared to those in low social risk group.

**Conclusion:**

Our findings suggest that a higher PsRS was associated with an elevated risk of incident rosacea in our study population.

## Introduction

Rosacea is a chronic inflammatory skin disease predominantly presenting on the convexities of the central face, which may manifest flushing, facial erythema, phymatous changes, papules and pustules, telangiectasia, and with ocular involvement (1).

The prevalence and incidence of rosacea vary geographically; however, it has been estimated by a systematic review of the published literature that 5.5% of the global population may be affected by rosacea (2). Recently, several population-based studies observed that prevalence of rosacea in Latin America, Asia, and Europe was all greater than 2% (3–5). Rosacea can lead to low self-esteem, anxiety, depression, and decreased social interactions, further adversely affects quality of life, social and mental health (6–8), and is associated with extra mean annual inpatient expenditures for mental health disorders of more than $2 million in the United States (9). According to a Chinese report, approximately 53.9 and 58.1% of Chinese rosacea patients affect anxiety and depression, respectively (10).

The exact pathogenesis of rosacea is unclear. Neurovascular dysregulation and neurogenic inflammation are thought to be a key pathogenic role in rosacea, in which epidermis and nerves interact and release substances for reciprocal sustenance thus promoting the development of rosacea (11, 12). The close relationship between stress and rosacea has been widely discussed and accepted (12–14). Psychological stress may lead to aberrant barrier homeostasis, stratum corneum integrity, and epidermal innate immunity (14) and is a potent activator of inflammation which can induce elevation of cytokines, activating both neuronal and inflammatory pathways (14–16). It has been suggested that psychogenic factors may be the aetiological factors for rosacea which are based on a rather outdated and small body of anecdotal evidence connecting life event stressors or other noticeable psychological distress with the onset of the skin disease (17). Recently, Spoendlin et al. did a matched case–control study which is the largest to date based on a high-quality, validated and large primary care database to access the association between psychogenic factors and incident rosacea, finding no altered relative risk of developing rosacea rising (17). However, this seemingly contradicts the potential pathogenesis of rosacea previously discovered. This may result from the limitation of the study that they quantified the contribution of the single psychogenic factors only, ignoring complex interconnection of social determinants of health such as individual factors, the wider social, community, physical environment factors, etc. For example, psychogenic factors, combined with socioeconomic status, neighborhood, and living environment, have been found to be the factors affecting health outcomes recently (18). Different aspects of social determinants affect people heath in a cumulative way (19), however, to our knowledge, there are no studies conducted to overall evaluate the impact of these social determinants on rosacea.

Therefore, considering this, we developed a polysocial risk score (PsRS) to assess participant-level social risk of rosacea, instead of determining the precise contribution of each social factor. With regard to polygenic risk score, a certain disease is considered the aggregate result of various interacting genes. Analogous to polygenic risk score, in polysocial risk score, a certain disease is the aggregate result of interactions among different social determinants (19). The polysocial risk score model can be used to predict the risk between various combinations of social situations and a particular health outcome. This approach was recently applied to identify the impact of social environments on mortality (20). We aim to quantify the influence of social determinants on rosacea comprehensively and investigate associations between the PsRS with the risks of incident rosacea.

## Methods

### Study design and population

We conducted a population-based prospective cohort study from January 2018 to December 2021 in five cities of Hunan province of China, including Changsha, Huaihua, Zhuzhou, Hengyang, and Changde. Totally, 11,523 government employees were recruited into the study. The baseline survey was conducted by a questionnaire collecting information on demographic, socioeconomic, and lifestyle behaviors, and the skin conditions of participants were assessed by the dermatologists in a dermatological physical examination at the same time. The skin health status of participants was reassessed every year since the enrolment of study during the follow-up period.

The follow-up began on the date of return of the questionnaire or the first physical examination. Participants contributed person-time from baseline to the date of the first diagnosis of rosacea during follow-up or the date of the last physical examination (due to loss to follow up or the end of the study), whichever came first.

In this study, only participants who have completed at least two consecutive dermatological physical examinations (*n* = 3,773) were included in the analysis. The study procedures were approved by ethics committee of Xiangya School of Public Health, Central South University (XYGW-2016-10).

### Covariates

Demographic characteristics included age and sex and lifestyle characteristics included alcohol drinking status, smoking status, frequency of sunbath, and frequency of physical exercise were all collected by questionnaires at baseline. New variables were created when including smoking and drinking for adjustments in multivariable models by further including information on daily average consumption of cigarettes and frequency of drinking per week in current smokers and drinkers. The body mass index (BMI; kg/m^2^) was calculated by dividing body weight in kilograms by height in meters squared.

### Ascertainment of rosacea

The diagnosis of rosacea was made by certified dermatologists at local tertiary hospitals. Dermatologists asked participants about their clinical signs, disease history, and family history. Then physical examinations were conducted to make the accurate diagnosis according to the diagnostic criteria issued by the National Rosacea Society Expert Committee in 2017 (at least one diagnostic or two major phenotypes) (1).

### Calculation of PsRS

Guided by previous literatures (18, 21, 22), we classified nine social determinants of health into three domains (socioeconomic status, psychosocial factors, and living environment) to capture the overall individual-level social risk. For socioeconomic status, when the participants met the following criteria (1) the annual household income was <100,000 Chinese Yuan (low household income; bellow median); (2) the education level was lower than undergraduate degree (low education level; bellow median), they were considered at risk. For psychosocial factors, when the participants met the following criteria (1) dine out in social gatherings less than once a week (social inactivity); (2) were ever misunderstood, blamed, or framed by others within last year (lack of social support); (3) had possible major depressive disorder or generalized anxiety disorder: screening positive for two-item Patient Health Questionnaire (PHQ-2 ≥ 3) or two-item Generalized Anxiety Disorder (GAD-2 ≥ 3; potential psychiatric disorders) (23, 24); (4) had experienced illness, injury, bereavement, or stress within last year (emotional distress), they were considered at risk. For living environment, when the participants met the following criteria (1) their familial *per capita* living space was below the median (<40 m^2^ per person; constricted living space); (2) their type of housing was bungalow (poor housing quality); (3) they were exposed to dust or chemical pollution in their living environments (dust or chemical pollution), they were considered at risk. More details are described in Appendix S1. The dichotomised social determinants of health were used to calculate the PsRS (ranging from 0 to 9 and a higher PsRS indicating greater social vulnerability). Besides, we roughly divided participants into four groups by approximately quartiles of PsRS: Q1 (0–1), Q2 (=2), Q3 (=3), and Q4 (≥4).

### Statistical analysis

Participants’ characteristics at baseline were summarized by categories of PsRS. Continuous variables were presented as mean ± standard deviation (SD), whereas categorical variables were presented as number and percentages (%). Between-group differences were compared using one-way ANOVA or chi-square test when appropriate. We used binary logistic regression models to analyze the associations between each individual social determinant of health and incidence of rosacea. The adjusted odds ratio (aOR) with corresponding 95% confidence interval (CI) was calculated. The incidence rate (per 1,000 person-years) was also calculated for each individual social risk factor. Then, we investigated the associations between PsRS and incidence of rosacea. PsRS was treated as a continuous variable in order to obtain a *p* value for trend. The potential non-linear relationship between PsRS and incidence of rosacea was examined by natural cubic splines. In addition, we conducted a subgroup analysis for the association of PsRS with incident rosacea by covariates to check the influence of various factors on the effect size of PsRS.

As for sensitivity analysis, first, we tried to minimize the effect of misdiagnosis introduced by several other disfiguring facial dermatoses (1), by excluding participants with prevalent or incident acne vulgaris, contact dermatitis, or seborrheic dermatitis. Besides, we further included inadequate exercise (exercise less than once a week) as a social determinant of health in calculating the PsRS in accordance with the Healthy People 2030 Initiative. All basic models were adjusted by age and sex, and the fully adjusted models were further adjusted by BMI, cigarette smoking, alcohol drinking, sunbath, and physical exercise.

Those participants with missing data for calculation of the PsRS were removed while missing data on covariates were imputed by multiple imputation.

All statistical analyses were performed under R software (version 4.1.3). And the *p* value of less than 0.05 was considered significant.

## Results

### Baseline characteristics stratified by PsRS categories

In all, 2,993 participants were included in the primary analyses after excluding those without follow-up time (*n* = 68), with prevalent rosacea (*n* = 51), and with missing data associated with PsRS (*n* = 661; Supplementary Figure S1). During a median follow-up of 2 years (interquartile range: 2–3 years; 7,457 total person-years), 69 incident rosacea cases were identified (incidence rate: 9.25 per 1,000 person-years). Table 1 showed the baseline characteristics of participants stratified by PsRS categories. Eventually, PsRS in our population ranged from 0 to 7. Of the total 2,993 eligible participants, 20.8, 32.4, 26.7, and 20.1% of them were categorized into four groups from lowest to highest social risks according to their PsRS. Compared with participants at lowest social risk (Q1), those in the group with higher risk had low education level, annual household income, and exercised inadequately (all values of *p* < 0.05).

**Table 1:** Baseline characteristics of the study population stratified by categories of PsRS (*n* = 2,993).

Characteristics	Polysocial risk score				Missing value
	Q1 (0–1)	Q2 (=2)	Q3 (=3)	Q4 (≥4)	
Participants, *n* (%)	623 (20.8)	971 (32.4)	799 (26.7)	600 (20.1)	
Age (year), mean ± SD	41.12 ± 9.71	38.79 ± 9.04	37.90 ± 8.68	37.65 ± 9.35	
Female, *n* (%)	289 (46.4)	572 (58.9)	520 (65.1)	359 (59.8)	
BMI (Kg/m^2^), mean ± SD	23.87 (3.59)	23.48 (3.59)	23.18 (3.61)	23.36 (3.94)	1
Annual household income (CNY), *n* (%)					
<50,000	3 (0.5)	58 (6.0)	114 (14.3)	175 (29.2)	
50,000 ~ 100,000	21 (3.4)	189 (19.5)	269 (33.7)	268 (44.7)	
100,000 ~ 200,000	326 (52.3)	435 (44.8)	258 (32.3)	106 (17.7)	
>200,000	273 (43.8)	289 (29.8)	158 (19.8)	51 (8.5)	
Education level, *n* (%)					
High school and below	2 (0.3)	13 (1.3)	38 (4.8)	118 (19.7)	
Undergraduate degree	331 (53.1)	537 (55.3)	466 (58.3)	337 (56.2)	
Postgraduate degree and above	290 (46.5)	421 (43.4)	295 (36.9)	145 (24.2)	
Current smoking, *n* (%)	82 (14.8)	95 (10.2)	69 (9.0)	72 (12.7)	175
Current drinking, *n* (%)	125 (20.1)	88 (9.1)	69 (8.6)	58 (9.7)	
Frequency of physical exercise, *n* (%)					242
<1 time/week	220 (38.4)	397 (44.3)	375 (50.8)	295 (54.2)	
1–2 times/week	154 (26.9)	274 (30.6)	190 (25.7)	143 (26.3)	
≥3 times/week	199 (34.7)	225 (25.1)	173 (23.4)	106 (19.5)	

PsRS, polysocial risk score; CNY, Chinese Yuan; and SD: standard deviation.

### Individual social determinants of health and incident rosacea

Table 2 showed associations between each individual social determinant of health and incident rosacea. Among different social determinants of health, we found that low education level and constricted living space were related to an increased risk of incident rosacea in which the ORs were 2.22 (95%Cl: 1.05, 4.72; *p* = 0.038) and 1.72 (95%Cl: 1.05, 2.82; *p* = 0.030), respectively.

**Table 2:** Association of individual social determinant of health with incident rosacea (*n* = 2,993).

Social determinant of health	No. of participants at risk, *n* (%)	No. of cases of rosacea/person-years	Incidence rate (Per 1,000 person-years)	Univariate model	
				OR (95%CI)	*p*
Total		69/7457	9.25		
Socioeconomic status					
Low education level	171 (5.7)	8/407	19.66	2.22 (1.05, 4.72)	0.038
Low household income	1,097 (36.7)	26/2691	9.66	1.05 (0.64, 1.71)	0.858
Psychosocial factors					
Social inactivity	2,584 (86.3)	64/6441	9.94	2.05 (0.82, 5.13)	0.124
Lack of social support	257 (8.6)	9/645	13.95	1.62 (0.79, 3.30)	0.185
Potential psychiatric disorders	209 (7.0)	8/548	14.6	1.78 (0.84, 3.76)	0.134
Emotional distress	764 (25.5)	19/1890	10.05	1.11 (0.65, 1.90)	0.699
Living environment					
Constricted living space	1,475 (49.3)	43/3736	11.51	1.72 (1.05, 2.82)	0.030
Poor housing type	231 (7.7)	8/586	13.65	1.59 (0.75, 3.36)	0.226
Dust or chemical pollution	710 (23.7)	20/1847	10.83	1.32 (0.78, 2.24)	0.300

### Association of PsRS with incident rosacea

**Figure 1:**
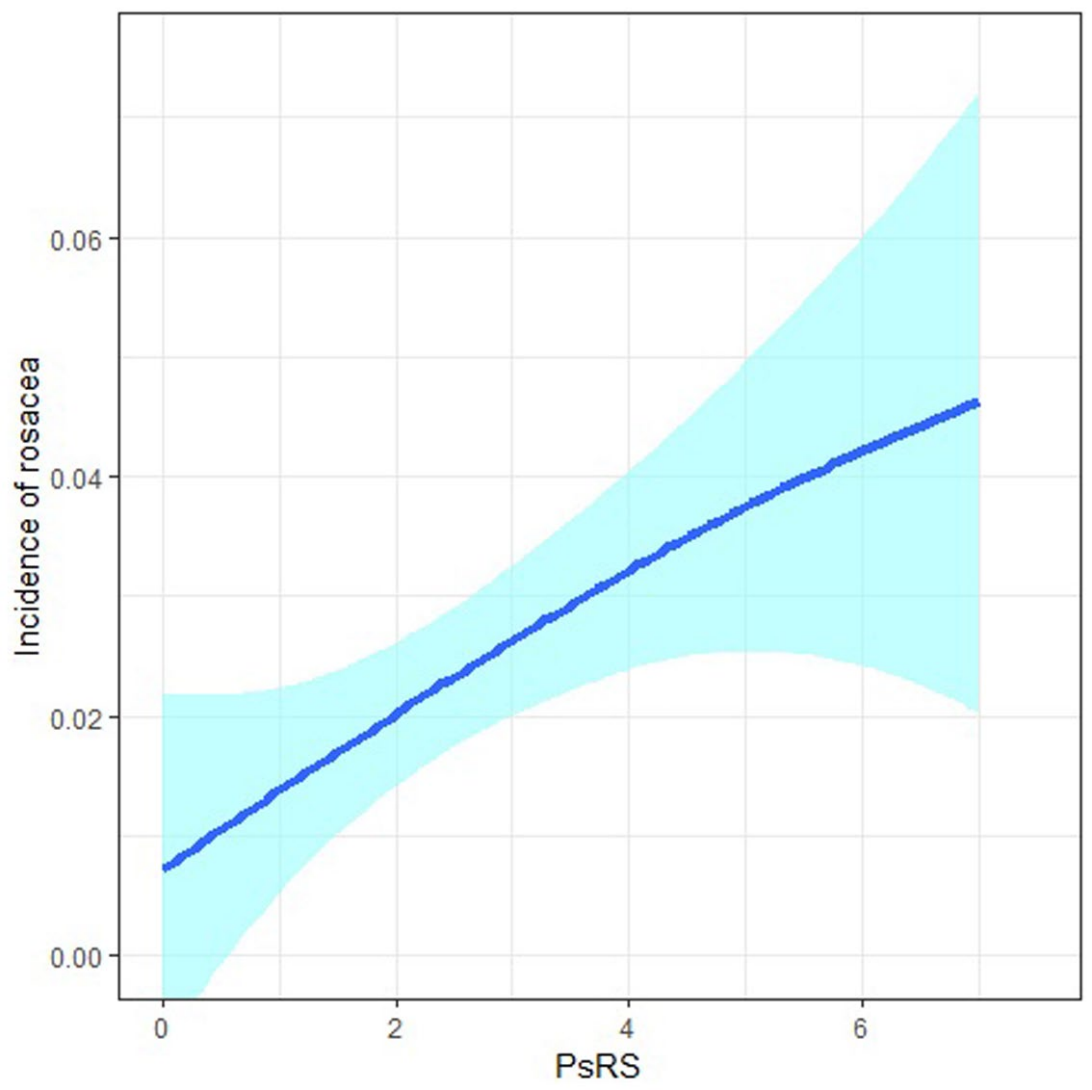
Positive non-linear relationship between PsRS and incidence of rosacea in cubic splines.

**Figure 2:**
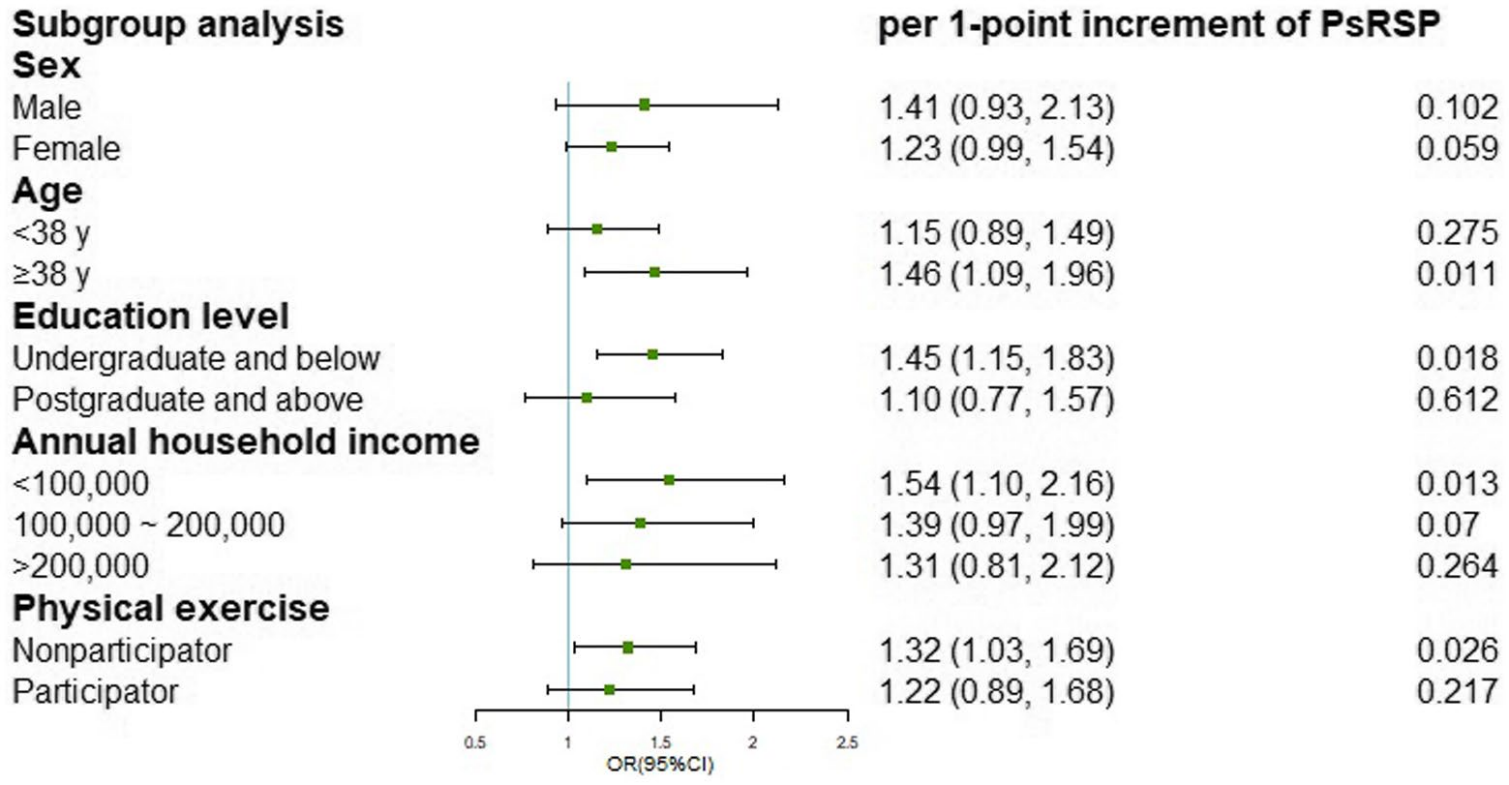
Subgroup analysis for the association of PsRS with incident rosacea by potential covariates.

After model adjustment, we found that participants with high social risk (Q4) had a significantly increased risk of developing rosacea with the aOR being 2.42 (95% CI: 1.06, 5.55), compared to those with low social risk (Q1; Table 3). Each point increment in PsRS was associated with a 29% (95%CI: 7,56) higher incidence of rosacea (*p*_trend_ = 0.008). Cubic splines showed a positive non-linear relationship between PsRS and the incidence of rosacea (Figure 1). Subgroup analysis for the association of PsRS with incident rosacea by covariates was presented in Figure 2. We observed a stronger effect size of PsRS among participants with lower levels of education, income, and inadequate exercise (nonparticipator).

**Table 3:** Association of PsRS with incident rosacea (*n* = 2,993).

	PsRS categories				*Per1-point increment of PsRS*	*p trend*
	Q1 (0–1)	Q2 (=2)	Q3 (=3)	Q4 (≥4)		
No. of cases of rosacea/person-years	8/1513	18/2413	22/2026	21/1505		
Incidence rate (per 1,000 person-years)	5.29	7.46	10.86	13.95		
Age- and sex-adjusted OR (95% CI)	Ref	1.27 (0.55, 2.95)	1.80 (0.79, 4.09)	2.42 (1.06, 5.55)	1.31(1.09,1.58)	0.004
Multivariate adjusted OR (95% CI)^*^	Ref	1.27 (0.54, 2.97)	1.77 (0.77, 4.06)	2.30 (1.00, 5.32)	1.29(1.07,1.56)	0.008

* Models were further adjusted by BMI (continuous), cigarette smoking (never, past, or current smoking of 1–14, 15–20, or >20 cigarettes/day), alcohol drinking (rarely, past, or current drinking of 1, 2–4, or ≥5 times/week), sunbath (rarely, sometimes, or frequently) and frequency of physical exercise.

### Sensitivity analysis

In the sensitivity analysis, the results of the primary analysis remained stable when excluding participants with prevalent or incident acne vulgaris, contact dermatitis, or seborrheic dermatitis or further including inadequate physical activity when calculating the PsRS (Supplementary Tables S1, S2).

## Discussion

In this population-based prospective cohort study among 11,523 participants, a polysocial score was created by using nine social determinants of health from three domains to evaluate participant-level exposure to aggregate social risks. Our results showed that participants with a high PsRS (≥4) had an almost 2-fold higher risk of incident rosacea in comparison with those with a low PsRS (0–1).

A previous study indicated no aetiological association between pre-existent depression or other mood disorders and incident rosacea (17), and they did not control other social determinants of health such as socioeconomic status and living environment because of no recording in their cohort study. However, housing disadvantage has been confirmed to impact mental health later in life (25). Similarly, another study found no significant association between occupational environment or education level with rosacea (26). These may suggest that assessing the contribution of an individual factor in incident rosacea only, disregarding the interactions among various factors is rarely helpful due to its complexity of pathogenesis. Therefore, in our present study, we quantified the influence of social factors comprehensively by conducting a PsRS incorporating nine social determinants of health including three domains (namely socioeconomic status, psychosocial factors, and living environment). Our results showed that a higher PsRS was associated with a higher risk of incident rosacea. The participants of new-onset rosacea in high social risk groups (Q3, Q4) accounted for approximately 62% of our population. Our result may indicate that there are some complex interconnections in these social determinants of health with unclear mechanisms. Because rosacea is a common problem that is underdiagnosed, and the left untreated can lead to physical disfigurement and emotional distress (27), this newly discovered potential risk of incident rosacea may be informative to discern a high-risk population and further perform early detection and adequate treatment to improve quality of life in rosacea.

Numerous reputable scientific and public health organizations have acknowledged the importance of social factors in health, including structural factors such as socioeconomic and socioeconomic position and intermediary factors such as behavioral factors and psychosocial factors that these factors may have effects on the health of population (28). The mechanisms underlying the detected PsRS–rosacea associations are not completely understood. Subordinate social status (peer disrespect or being regarded as less important or competent) and psychosocial stress had been found to be associated with increased pro-inflammatory cytokines (14, 29) and cortisol is often increased when people are persistently lacking in social status or may soon lose it (30). Cytokines as well as chemokines mediate leukocyte recruitment, activate distinct leukocyte subsets to orchestrate an inflammatory response, and induce the characteristic histopathological features of rosacea (31). Cortisol increases persistently may lead to compromised permeability barrier homeostasis, stratum corneum cohesion, wound healing, and epidermal innate immunity in normal skin (14). However, due to multiple clinical manifestations of rosacea, it may involve complex pathobiology through different regulatory systems (32). This only provides a partial explanation. Though mechanisms remain unknown, our results suggest that incident rosacea increases under the combined effects of social determinants of health.

In our analysis of nine individual social determinants of health and incident rosacea, low education level was determined to be significantly associated with the onset of rosacea in which the aOR was 2.22. Our finding appears to be inconsistent with finding of the study by Abram et al. (26). We therefore believe that our finding requires cautious interpretation. Our study population, the government employees, generally have higher education level and more spacious housing space compared with other groups which may result in population selection bias. And in our study, we had a high rate of loss to follow-up of the population and this population had the characteristics of lower education level (Supplementary Table S3). Moreover, constricted living space may indicate household overcrowded. Crowding is associated with several measures of socioeconomic deprivation including low income and low education level, and is revealed to be related to poor self-rated health and mental health (33–35). Though we also found that constricted living space was significantly associated with increased incident rosacea, this may result from our population selection bias. Despite these, we found no significant associations for other seven social individual determinants of health but PsRS, which illustrated that these factors influence health in complex and interrelated ways.

This study had several strengths. We are the first to jointly evaluate the impact of multiple domains of social risk factors including socioeconomic status, psychosocial factors, and living environment on incident rosacea. And this PsRS approach allows for a comprehensive estimation of participant-level exposure to social risks from various domains (18). However, several limitations of our study warrant attention. First, although we have controlled various confounders, the unmeasured confounding might still exist and the reverse causality was inevitable, thus was insufficient to draw a causality inference, even if the sensitivity analysis disclosed the robustness of our results. Second, the data about social factors were self-reported and collected only once, which may give rise to measurement errors. Repeated measurements are needed to catch long-time varieties in PsRS. Third, our study had a high rate of loss to follow-up (referring to those excluded for only attending skin examination at baseline) and this population had lower education level and income (Supplementary Table S1). And our study population was government employees which have a higher education level and more spacious housing space compared to other occupations in China, thus, the selection bias was inevitable even if we had adjusted the models. Forth, the PsRS was created by counting 9 different social risk factors, presuming that each factor has the same impacts on health, which may be at variance with reality.

In conclusion, our results suggest that the PsRS determined by using nine social determinants of health, was associated with an increased incidence of rosacea in our study population.

## What is already known about this topic?

- The associations between a single risk factor with incident rosacea have been described previously.

## What does this study add?

- We quantified the influence of social factors comprehensively by conducting a PsRS incorporating nine social determinants of health including various domains and found a high PsRS was associated with an elevated risk of incident rosacea in our study population.

## What are the clinical implications of this work?

- This newly discovered potential risk of incident rosacea may be informative to discern a high-risk population and further perform early detection and adequate treatment to improve quality of life in rosacea.

## Data availability statement

The raw data supporting the conclusions of this article will be made available by the authors, without undue reservation.

## Ethics statement

The studies involving human participants were reviewed and approved by the institutional research ethics boards of Xiangya School of Public Health, Central South University (XYGW-2016-10). The patients/participants provided their written informed consent to participate in this study.

## Author contributions

MS and JL contributed to conception and design of the study. PC performed the statistical analysis. ZY wrote the first draft of the manuscript. ZF, BW, YT, YX, XC, DL, and SX contributed to investigation. All authors contributed to the article and approved the submitted version.

## Funding

This work was supported by the National Key Research and Development Program of China (No. 2021YFF1201205) and the National Natural Science Funds for Distinguished Young Scholars (82225039).

## Conflict of interest

The authors declare that the research was conducted in the absence of any commercial or financial relationships that could be construed as a potential conflict of interest.

## Publisher’s note

All claims expressed in this article are solely those of the authors and do not necessarily represent those of their affiliated organizations, or those of the publisher, the editors and the reviewers. Any product that may be evaluated in this article, or claim that may be made by its manufacturer, is not guaranteed or endorsed by the publisher.
