# Supplementary material for: Associations of polysocial risk score with incident rosacea: a prospective cohort study of government employees in China
Source: Front Public Health. 2023 May 3;11:1096687. doi: 10.3389/fpubh.2023.1096687 (PMC10191232; doi:10.3389/fpubh.2023.1096687)
Supplement: Supplementary file 1 [file Table_1.DOCX]

# PsRS Questionnaire

1. What is the annual income received by your household?

1) Less than 50,000 Chinese Yuan

2) 50,000-100,000 Chinese Yuan

3) 110,000-200,000 Chinese Yuan

4) 210,000-300,000 Chinese Yuan

5) 310,000-500,000 Chinese Yuan

6) 510,000-100,000 Chinese Yuan

7) Over 10,000,000 Chinese Yuan

2. Which of the following qualifications do you have?

1) Junior secondary school degree or below

2) Senior secondary school, secondary specialized school, or skilled Workers school degree

3) Undergraduate degree

4) Graduate degree

3. How many times in a week do you dine out in social gatherings?

1) less than once a week

2) 2 or 3 times a week

3) 4 or 5 times a week

4) more than 5 times a week

4. Have you experienced being misunderstood, blamed, or framed by others within last year

1) Yes

2) No

5. Over the last 2 weeks, how often have you been bothered by any of the following problems?

Give answers as 0 to 3, using this scale:

0=Not at all; 1=Several days; 2=More than half the days; 3=Nearly every day

a. Little interest or pleasure in doing things

1)0

2)1

3)2

4)3

b. Feeling down, depressed, or hopeless

1)0

2)1

3)2

4)3

6. Over the last 2 weeks, how often have you been bothered by any of the following problems?

Give answers as 0 to 3, using this scale:

0=Not at all; 1=Several days; 2=More than half the days; 3=Nearly every day

a. Feeling nervous, anxious or on edge

1)0

2)1

3)2

4)3

b. Not being able to stop or control worrying

1)0

2)1

3)2

4)3

7. Have you experienced any of the following:

Serious illness, injury or sault to yourself; illness, injury or assault of a close relative; death of a close relative; death of a spouse or partner; financial difficulties; marital separation/divorce?

1) Yes

2) No

10. What type of housing do you live in?

1) Bungalow

2) Apartment building

3) Villa

11. Including yourself, how many people live in your house? (numbers only)?

12. What is the size of your house (fill numbers only)?

13. Are you exposed to dust or chemical pollution in your living environments?

1) Yes

2) No

# Components of the polysocial risk score

**Socioeconomic status:**

Low household income：0 = annual household income is more than 100,000 Chinese Yuan; 1 = less than 100,000 Chinese Yuan

Low education level: 0 = highest education level is undergraduate degree or graduate degree; 1 = lower than undergraduate degree

**Psychosocial factors:**

Social inactivity: 0= dine out in social gatherings once a week or more often;1= less than once a week

Lack of social support: 1 = have experienced being misunderstood, blamed, or framed by others within last year; 0 = none

Potential psychiatric disorders: 1= screen positive for Patient Health Questionnaire-2 (PHQ-2 ≥3) or Generalized Anxiety Disorder 2-item (GAD-2 ≥3); 0=none

Emotional distress: 1 = have experienced illness, injury, bereavement, stress within last year; 0 = none

**Living environment:**

Constricted living space: 0= above the median of familial per capita living space (40 m2 per person); 1=below the median

Poor housing quality: 0= live in apartment building or villa;1= live in bungalow

Dust or chemical pollution: 1= being exposed to dust or chemical pollution in their living environments; 0=none

**Table S1. Sensitivity analysis by excluding participants with prevalent or incident acne, contact dermatitis, or seborrheic dermatitis (N=270)**

|  | PsRS categories | | | | *P-trend* |
| --- | --- | --- | --- | --- | --- |
|  | Q1 (0-1) | Q2 (=2) | Q3 (=3) | Q4 (≥4) |  |
| No. of Cases of Rosacea/Person-Years | 8/1390 | 14/2191 | 18/1880 | 20/1381 |  |
| Incidence Rate (Per 1000 Person-years) | 5.76 | 6.39 | 9.57 | 14.48 |  |
| Age- and sex-adjusted OR (95% CI) | Ref | 1.00 (0.41, 2.41) | 1.47 (0.63, 3.43) | 2.35 (1.02, 5.42) | 0.004 |
| Multivariate adjusted OR (95% CI) ^*^ | Ref | 0.99 (0.41, 2.40) | 1.42 (0.60, 3.35) | 2.22 (0.95, 5.18) | 0.007 |

^*^: Models were further adjusted by BMI (continuous), cigarette smoking (never, past, or current smoking of 1–14, 15–20, or >20 cigarettes/d), alcohol drinking (rarely, past, or current drinking of 1, 2-4, or ≥ 5 times/week), sunbath (rarely, sometimes, or frequently) and frequency of physical exercise.

|  | PsRS categories | | | *P-trend* |
| --- | --- | --- | --- | --- |
|  | Q1 (0-2) | Q2 (=3) | Q3 (≥4) |  |
| No. of Cases of Rosacea/Person-Years | 13/2890 | 24/2061 | 32/2506 |  |
| Incidence Rate (Per 1000 Person-years) | 4.5 | 11.64 | 12.77 |  |
| Age- and sex-adjusted OR (95% CI) | Ref | 2.45 (1.23, 4.88) | 2.69 (1.39, 5.21) | 0.001 |
| Multivariate adjusted OR (95% CI) ^*^ | Ref | 2.42 (1.19, 4.90) | 2.51 (1.23, 5.14) | 0.007 |

**Table S2. Sensitivity analysis by further including inadequate exercise in calculating PsRS (n=2993)**

^*^: Models were further adjusted by BMI (continuous), cigarette smoking (never, past, or current smoking of 1–14, 15–20, or >20 cigarettes/d), alcohol drinking (rarely, past, or current drinking of 1, 2-4, or ≥ 5 times/week), and sunbath (rarely, sometimes, or frequently).

**Table S3. Baseline characteristics comparison between the study population and those lost to follow up**

| Characteristics | Study population (N=3773) | Lost to follow up (N=7750) | P-value |
| --- | --- | --- | --- |
| Female, n (%) | 2243 (59.4) | 4567 (58.9) | 0.608 |
| Age (year), mean ± SD | 38.65 ± 9.22 | 39.18 ± 11.73 | 0.018 |
| BMI (Kg/m2), mean ± SD | 23.46 ± 3.79 | 23.79 ± 5.14 | <0.001 |
| Annual household income (CNY), n (%) |  |  | <0.001 |
| <50,000 | 438 (12.1) | 1171 (17.3) |  |
| 50,000 ~ 100,000 | 923 (25.6) | 2080 (30.7) |  |
| 100,000 ~ 200,000 | 1339 (37.1) | 2280 (33.6) |  |
| >200,000 | 908 (25.2) | 1255 (18.5) |  |
| Education level, n (%) |  |  | <0.001 |
| High school and below | 189 (5.9) | 512 (8.6) |  |
| Undergraduate degree | 1801 (56.1) | 3973 (66.7) |  |
| Postgraduate degree and above | 1225 (38.1) | 1472 (24.7) |  |
| Smoking status, n (%) |  |  | 0.126 |
| Non-smoker | 2967 (86.9) | 5960 (85.6) |  |
| Current smoker | 377 (11.0) | 863 (12.4) |  |
| Past smoker | 71 (2.1) | 136 (2.0) |  |
| Alcohol drinking, n (%) |  |  | 0.547 |
| Non-drinker | 3182 (88.2) | 6351 (88.2) |  |
| Current drinker | 392 (10.9) | 767 (10.6) |  |
| Past drinker | 34 (0.9) | 84 (1.2) |  |

CNY: Chinese Yuan; SD: standard deviation.
