# Supplementary figures and images for: Associations of polysocial risk score with incident rosacea: a prospective cohort study of government employees in China
Source: Front Public Health. 2023 May 3;11:1096687. doi: 10.3389/fpubh.2023.1096687 (PMC10191232; doi:10.3389/fpubh.2023.1096687)

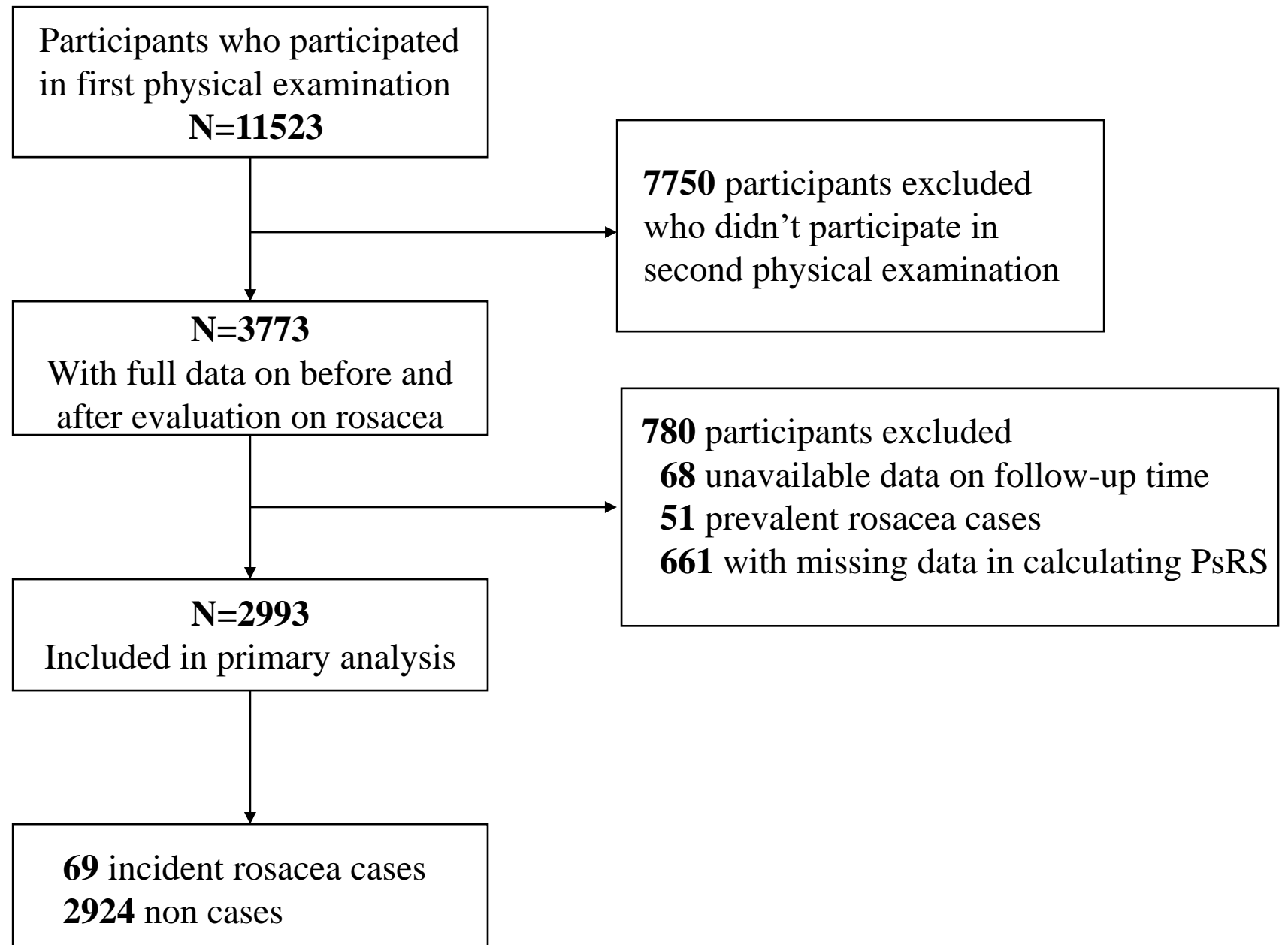

**Figure S1. Study flow chart**

Supplement: Supplementary file 2 [file Data_Sheet_1.PDF]
